# An "unexpected" role for EMT transcription factors in hematological development and malignancy

**DOI:** 10.3389/fimmu.2023.1207360

**Published:** 2023-08-03

**Authors:** Karthika Radhakrishnan, Lynda Truong, Catherine L. Carmichael

**Affiliations:** ^1^ Centre for Cancer Research, Hudson Institute of Medical Research, Clayton, VIC, Australia; ^2^ Monash University, Faculty of Medicine, Nursing and Health Sciences, Clayton, VIC, Australia

**Keywords:** EMT, hematopoiesis, leukemia, blood cells, stem cells, malignancy

## Abstract

The epithelial to mesenchymal transition (EMT) is a fundamental developmental process essential for normal embryonic development. It is also important during various pathogenic processes including fibrosis, wound healing and epithelial cancer cell metastasis and invasion. EMT is regulated by a variety of cell signalling pathways, cell-cell interactions and microenvironmental cues, however the key drivers of EMT are transcription factors of the ZEB, TWIST and SNAIL families. Recently, novel and unexpected roles for these EMT transcription factors (EMT-TFs) during normal blood cell development have emerged, which appear to be largely independent of classical EMT processes. Furthermore, EMT-TFs have also begun to be implicated in the development and pathogenesis of malignant hematological diseases such as leukemia and lymphoma, and now present themselves or the pathways they regulate as possible new therapeutic targets within these malignancies. In this review, we discuss the ZEB, TWIST and SNAIL families of EMT-TFs, focusing on what is known about their normal roles during hematopoiesis as well as the emerging and “unexpected” contribution they play during development and progression of blood cancers.

## 1 Introduction

The Epithelial to Mesenchymal Transition (EMT) is a physiological process whereby epithelial cells transform into a more mesenchymal phenotype, enabling them to migrate away from their epithelial layer of origin. Typically, epithelial cells are arranged side by side through strong intercellular junctions and are attached to the basement membrane with a clear apical-basal polarity. The cells are held together and to the basement membrane through various cell adhesion molecules such as claudin and E-cadherin. In contrast, mesenchymal cells are generally motile with only transient polarity and intercellular junctions. Depending on the biological context, EMT can be classified into three types. Type I EMT occurs during normal embryonic development and was first described in chicken embryos (1, 2), Type II EMT occurs during tissue repair, wound healing and fibrosis (reviewed in (3, 4) and Type III EMT occurs during pathogenic processes – most notably cancer metastasis (5).

Several key transcription factors, hereafter termed EMT-transcription factors (EMT-TFs), play fundamental roles in regulating the initiation and progression of all three types of EMT. These EMT-TFs belong to three distinct families, the ZEB (ZEB1 and ZEB2), TWIST (TWIST1 and TWIST2) and SNAIL (SNAI1, SNAI2 and SNAI3) families. During embryogenesis, these EMT-TFs are critically important for regulating essential developmental processes such as gastrulation, mesoderm specification, neural crest formation and skeletal development (6–11). In the malignant context, EMT-TFs also play fundamental roles in critical aspects of cancer cell function and survival including tumor progression and metastasis, resistance to therapy, immune evasion and stemness (12–18).

There has been an increasing interest in the role EMT-TFs play in the development and functioning of the hematopoietic system, even though there is no obvious EMT process involved. Even more surprisingly, these EMT-TFs are now also emerging as significant contributors to the pathogenesis and development of malignant hematological disease. However, the underlying mechanisms of their involvement are not yet fully understood. In this review, we discuss the ZEB, TWIST, and SNAIL families of EMT-TFs and outline their “unexpected” functions in regulating normal and malignant blood cell development.

## 2 EMT transcription factors (EMT-TFs)

### 2.1 ZEB family

The Zinc-finger E-box binding homeobox (ZEB) family of transcription factors were first discovered in *Drosophila melanogaster* by Fortini et al. (19). Fortini described two highly conserved homologous genes, *zfh1* and *zfh2 (*now known as *ZEB1* and *ZEB2)* that encode for large proteins containing multiple N- and C- terminal DNA-binding C_2_H_2_ zinc-fingers separated by a homeodomain region (19). Lai et al. found z*fh1* to be expressed in the early embryonic mesoderm, along the dorsal vessel and in the developing central nervous system (CNS). Expression of *Zfh2*, on the other hand, was largely localized to the CNS and hindgut of developing embryos (20). Chicken *Zeb1 (Zfh1)* was later identified during embryonic lens development as a transcriptional repressor of the δ1-crystallin enhancer core (21). This study subsequently found ZEB1 to be primarily expressed during the post-gastrulation period in mesodermal tissues, neuroectoderm, neural crest and lens (21). Murine *Zeb1* was first cloned from a mouse brain cDNA library in 1996 (22), while mouse *Zeb2* was initially named *Sip1* (for SMAD-interacting protein 1) following its identification in a yeast two-hybrid system using the MH2 domain of Xenopus Smad1 as bait (23).

The vertebrate ZEB1 and ZEB2 proteins share a high degree of structural similarity, with both carrying C_2_H_2_ zinc-finger clusters at their N- and C-terminal ends that bind E-box and E-box-like DNA motifs (5’-CACCTG-3’) (23, 24). Around 85% of protein sequence identity within ZEB1 and ZEB2 is shared at the zinc-finger domains, whereas only 30-50% sequence identity is shared in the intervening region containing the SMAD interaction domain (SID), homeodomain (HD) and C-terminal binding repressor protein (CtBP) interaction domain (CID) (25, 26). ZEB proteins primarily act as transcriptional repressors, through interaction with SMAD proteins, the CtBP and histone remodeling complexes such as the nucleosome remodeling and deacetylase complex (NURD) (27, 28). One of the best characterized targets of ZEB proteins is the CDH1 gene, encoding E-cadherin, a key epithelial gene that is downregulated during the EMT process (27, 29).

*Zeb1* knockout mice display skeletal and craniofacial defects and die shortly after birth due to a failure to respirate (30, 31). Homozygous *Zeb1* mutant mice, lacking the C-terminal zinc-finger domain, also experience perinatal lethality with ~80% of mice dying within two days of birth. However, in contrast to full knockout mice, *Zeb1* mutant mice are morphologically normal with the exception of a significantly reduced thymus (32). In the adult ZEB1 has been shown to be a critical regulator of bone development, with *Zeb1* expression found to be downregulated as mesenchymal stem cells (MSCs) differentiate down the osteoblastic lineages in the presence of BMP-2 (33). *In vitro* knockdown of *Zeb1* in MSCs resulted in enhanced osteogenesis, while *in vivo* osteoblast knockdown of *Zeb1* increased bone mass in the ovariectomized mouse model of osteoporosis (34). Interestingly, Fu et al. reported that *Zeb1* deletion in endothelial cells reduced bone associated angiogenesis and subsequently impaired bone formation (35). These findings indicate that ZEB1 has differential functions within endothelial and osteoblastic cells which coordinately contribute to bone development and maintenance. How the expression of ZEB1 is controlled in these different cell types and what level of crosstalk is involved remains to be elucidated.

*Zeb2* KO mice die around E9.5, exhibiting growth retardation as well as failure of neural tube closure and neural crest delamination (36). Various conditional *Zeb2* deletion models have demonstrated a critical role for ZEB2 in neurological, gastrointestinal, craniofacial and CNS development (reviewed in (37). Germline *de novo ZEB2* mutations or deletions cause a dominant syndromic form of Hirschsprung disease (HSCR) called Mowat-Wilson Syndrome. Patients with this syndrome exhibit microcephaly, mental retardation, submucous cleft palate among other distinct facial features (38–40).

### 2.2 TWIST family

The Twist family consists of two members, TWIST1 and TWIST2 (DERMO-1), which exist as a sub-class of the basic helix-loop-helix (bHLH) superfamily of transcriptional repressors. This superfamily is characterized by the presence of a bHLH motif, which is a short chain of basic amino acids followed by two amphipathic α-helices separated by a more divergent loop (41–43). The basic region of the bHLH motif serves to recognize and bind E-box sequences in the DNA, while the HLH region is responsible for forming homo/heterodimers with other bHLH proteins (44, 45). Through recognition of distinct E-box sequences, heterodimerization with different bHLH proteins allows significant heterogeneity in the target DNA sequences bound by TWIST proteins.

*Twist1* was first discovered in *Drosophila melanogaster* by Simpson et al. who identified that embryonic lethal *twi* mutations resulted in abnormal gastrulation, impaired dorso-ventral patterning and failed mesoderm differentiation, resulting in an embryo with a ‘twisted’ phenotype (46). The Drosophila *twist* gene was subsequently cloned in 1987 (7) and mouse *Twist1* in 1991 (47). *Twist1* KO mouse models are embryonic lethal at E11.5, and show a failure of neural tube closure and developmental defects impacting somite formation, cranial mesenchyme and limb development (10). The human *TWIST1* gene displays 92% sequence identity with murine *Twist1* and was mapped by Wang et al. to chromosome 7p21 (48). Haploinsufficiency of the *TWIST1* gene in humans results in Saethre-Chotzen syndrome, a congenital anomaly characterized by craniosynostosis as well as facial and limb anomalies (49–51).

The *Twist2* gene was first discovered in mice by Li et al. using a yeast two-hybrid system to screen for binding partners of the bHLH protein, E12. This study identified a novel bHLH dimerization partner, which was named *Dermo-1* due to its expression in the embryonic murine dermis (52). Like TWIST1, TWIST2 is also detectable throughout embryonic development and during the neonatal period, however it is downregulated in adult tissues (52). An early study by Sosic et al. revealed that unlike *Twist1* KO, *Twist2* KO mice were viable and born at expected mendelian ratios. *Twist2* KO mice did, however, display significant post-natal abnormalities including growth retardation, cachexia and elevated levels of pro-inflammatory cytokines. KO mice also experience perinatal lethality with 60% of homozygous KO mice dying within three days of birth (53). A later study by the same group identified germline nonsense homozygous mutations in the *TWIST2* gene in patients with autosomal recessive Setleis syndrome, an inherited developmental disorder under the branch of Focal Facial Dermal Dysplasia (FFDD) (54, 55).

### 2.3 SNAIL family

The Snail family of transcription factors consists of three members, SNAI1 (Snail), SNAI2 (Slug) and SNAI3 (Smuc) characterized by the presence of a highly conserved C_2_H_2_ zinc-finger C-terminal region containing four to five zinc fingers and a more diverse amino-terminal region. The C_2_H_2_ zinc-fingers allow Snail family transcription factors to recognize and bind E-box elements in target DNA sequences (56, 57). All Snail family members also contain a highly conserved eight amino acid (MPRSFLVK) N-terminal SNAG repressor domain (58, 59). Studies have shown that the Snail family predominantly act as transcriptional repressors across a plethora of developmental and EMT-related pathways (60–62).

*SNAI1* was the first and founding member of the Snail family, originally identified in *Drosophila melanogaster.* Embryos with loss of function mutations in the *Sna* gene show defects in gastrulation, mesoderm specification and embryo patterning resulting in an embryo resembling a Snail (46, 63). The murine *Snai1* gene was cloned in 1992 and found to be expressed in mesoderm and primitive ectoderm during gastrulation, as well as in the pre-somitic mesoderm, neural crest, developing lung, gut and kidney and early stages of cartilage differentiation (64). Mouse *Snai1* KO is embryonically lethal at E7.5-8.5 due to defects in gastrulation and mesoderm formation (65). SNAI1 is a major driver of the EMT process, playing a key role in repressing the epithelial specific cadherin, E-Cadherin, through binding to E-box sequences in its promoter (66). Other EMT related genes regulated by SNAI1 include epithelial markers such as claudins, occludins and desmoplakins and mesenchymal markers such as vimentin and fibronectin (60, 67, 68).

The *Snai2* gene, also known as *Slug*, was first identified by Nieto et al. in chickens as a homolog for the Xenopus *snai1* gene (69). Using antisense oligos towards *snai2*, Nieto et al. further identified a role for this gene in EMT processes associated with neural tube development and mesoderm emergence from the primitive streak (69). The mouse homolog of *Snai2* was subsequently cloned from mouse cDNA using chicken *Snai2* oligos, and found to initiate EMT when ectopically expressed in a rat carcinoma cell line (70). In sharp contrast to *Snai1* KO mouse models, *Snai2* KO mice are viable however, exhibit severe growth retardation and eyelid malformations as well as pigmentation, gonadal and hematopoietic defects post birth (71, 72). Germline homozygous SLUG deletions have been identified in Waardenburg disease, a congenital disorder characterized by hearing loss and pigmentation changes in hair, skin and eyes (73).

The third member of the Snail superfamily, *Snai3* (also known as *Smuc*), was the last to be identified and is the least well understood. In 2000, Kataoka et al. isolated a *Snai1*-related gene from mouse tissues, initially named *Smuc*, which was highly expressed in the skeletal muscle and thymus (74). The human *SNAI3* gene was later identified using *in silico* analysis, and determined to contain the conserved SNAG domain as well as five DNA-binding zinc fingers (75). Murine *Snai3* KO mice do not exhibit any obvious abnormalities [Bradley et al., 2013 (76); Pioli et al., 2013 (77)], suggesting a possible redundant role for *Snai3* alongside its other family members.

## 3 An emerging role for EMT-TFs in hematopoiesis

**Figure 1:**
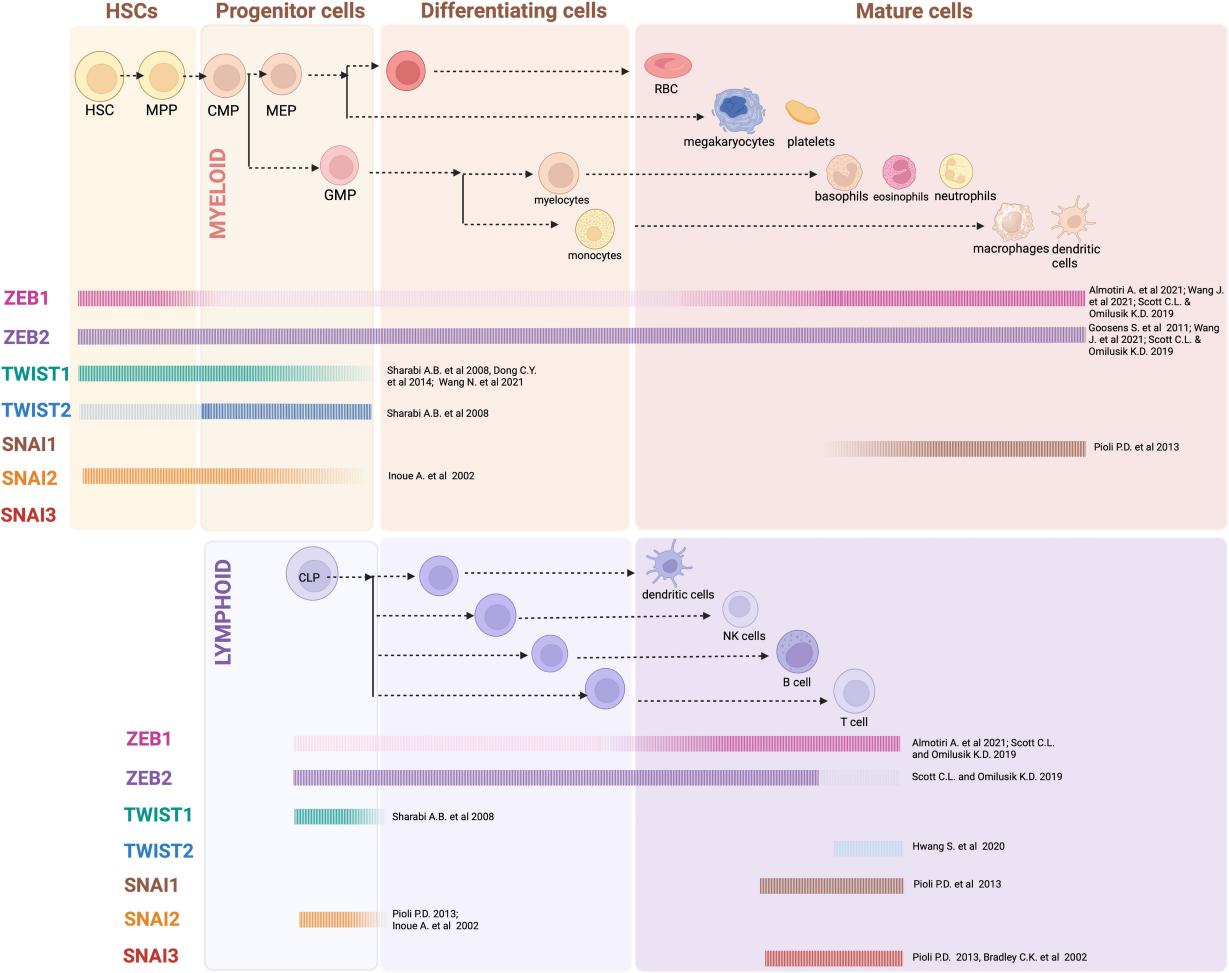
EMT-TF expression in hematopoiesis: Generalized overview of EMT-TFs expression throughout the hematopoietic hierarchy, as outlined in referenced articles. In many cases expression of EMT-TFs has not been thoroughly assessed experimentally, and current knowledge relies on gene expression datasets obtained from sorted mouse and/or human cells. Created with BioRender.com.

Hematopoiesis is not readily associated with EMT, although an exception to this could be the emergence and generation of primitive HSCs in the embryo (78). In vertebrates, hematopoiesis occurs in two waves: primitive hematopoiesis, which occurs during early embryogenesis, and definitive hematopoiesis, which occurs during later stages of development (79, 80). Unlike primitive HSCs, definitive HSCs can give rise to the entire hematopoietic system and persist throughout life. Definitive HSCs arise from a population of hemogenic endothelial cells in the embryonic AGM (embryonic aorta, gonad and mesonephros region) (81). This process of Endothelial to Hematopoietic transition (EHT) closely resembles EMT and is characterized by a loss of endothelial characteristics and increased migratory capabilities (82, 83). EMT-TFs have thus far not been implicated in EHT and HSC emergence in the embryo, however there is increasing evidence that these factors are expressed in hematopoietic cells and play important roles in regulating normal blood cell development and function (**Figure 1**; **Tables 1**, **2**).

**Table 1:** Summary of EMT-TF knockout or mutant mouse model hematopoietic phenotypes.

KNOCKOUT/DEFICIENCY						
GENE	MODEL	STEM and PROGENITOR	MYELOID	LYMPHOID	OTHER PHENOTYPE	REFERENCE
ZEB1	Constitutive C-terminal deletion			Reduced thymus size, reduced T-cells in thymus and periphery. Majority T cells detected in thymus were mature DP and SP	CD4 + ATLL	Higashi Y 1997 (32), Hidaka T 2008 (84)
	Constitutive C-terminal deletion			Reduced thymus size with reduced DF T-cells but enhanced DN and mature SP T cells. Normal T cells but abnormal B cells in the spleen		Arnold C, 2012 (85)
	Inducible KO (MX1-Cre)	Normal HSC numbers at steady state but have defective competitive transplantation ability, reduced self-renewal and impaired differentiation. Normal HSC homing and migration	Reduced monocytes, normal numbers of granulocytes	Normal lymphoid cells in blood Reduced thymus size with reduced DP T-cells but enhanced DN and mature SP T cells. DN1 to DN2/3 block also evident Reduced effector and central memory CD8+ T cells in periphery		Almotiri A, 2021 (100)
	Conditional KO (haematopoietic cells): inducible KO (RosaERT2Cre)	Reduced HSPC number	Reduced monocytes	Reduced thymic cellularity, reduced % DN4 with increased CD8+. B-cells normal		Wang J, 2021 (86)
ZEB2	Constitituve KO	Impaired HSPC differentiation all lineages and migration in embryos				Goossens S, 2011 (87)
	Inducible KO (MX1- Cre)	Increased HSCs and MEPs. but reduced GMPs	Reduced monocytes and erythroid cells, enhanced granulocytes and immature megakaryocytes	Reduced B-cells and a block in development from pre-pro-B to pro- B. Reduced T cells	Myeloproliferative disease splenomegaly bone marrow fibrosis	Li J, 2017 (88)
TWIST1	Constitutive KO	Reduced GM-CFU. M-CFU, BFU-E formed from AGM 10.5		E10.5 AGM cells show impaired B cell development on OP9 co-culture		Kulkeaw K, 2017 (89)
	Constitutive KO	HSCs have reduced repopulating capacity				Dong CY, 2014 (90)
	Inducible KO (MX1- Cre)	Reduced HSPCs with impaired self-renewal and reduced quiescence. Reduced lymphoid and meg/eryth differentiation with enhanced granulocyte/macrophage differentiation. Loss of lymphold biased HSCs. Reduced engraftment in competitive transplantation. HSC homing normal			HSCs sensitive to irradiation induced DNA damage and apoptosis. 5FU treatment also led to rapid HSC exhaustion and haematopoletic failure	Wang J, 2021 (91)
TWIST2	Constitutive KO	Enhanced GMPs with increased proliferative capacity and enhanced differentiation *in vitro*	Enhanced myeloid cell numbers, increased macrophages, neutrophils and basophils. Normal erythrocytes and platelets	Normal lymphoid numbers	Meylodysplasia/ myeloproliferation?	Sharabi A B, 2008 (92)
SNAI1	Conditional haematopoietic specific KO		Normal	Normal		Carmichael C, 2017 (93)
SNAI2	Constitutive KO	Normal HSC numbers. Reduced BFU-E and CFU-E in spleen, normal in BM	Normal myeloid cell numbers macrocytic anaemia	Reduced thymus size, reduced DP T cells. B cells normal increased T cell apoptosis	Stress erythropoesis impaired	Perez-Losada J, 2002 (72)
	Constitutive KO	Slightly reduced CFU-GM CFU-M, BFU-E CFU-E	Normal myeloid cell numbers	Normal lymphoid numbers	HSCs increased sensitiviy to DNA damage and increased apoptosis, unable to recover haemat system after irradiation LD50 dose.	Inoue A, 2002 (94)
	Constitutive KO	HSCs show enhanced repopulating capacity in competitive transplants. HSCs show normal homing and differentiation but increased self-renewal and proliferation capacity	Normal	Normal	5FU induced enhanced HSC cycling and proliferation leading to enhanced haematopoietic recovery	Sun Y, 2010 (95)

**Table 2:** Summary of EMT-TF overexpression mouse model hematopoietic phenotypes.

OVEREXPRESSION						
GENE	MODEL	STEM and PROGENITOR	MYELOID	LYMPHOID	OTHER PHENOTYPE	REFERENCE
ZEB1	Transgenic mouse, Vav-iCre		Expanded monocytic development, increased myeloid, extramedullary haematopoiesis, splenomegaly			Wang J, 2021 (86)
ZEB2	Transgenic mouse, Vav-iCre		Expanded monocytic development, increased myeloid, extramedullary haematopoiesis, splenomegaly	Impaired T cell development, DN block, expanded DN population	ETP-ALL, extramedullary haematopoiesis, splenomegaly, myeloproliferation?	Wang J, 2021 (86)
TWIST1	Retroviral overexpression and transplant	Enhanced quiescnece and self renewal, enhanced repopulating capacity, myeloid-erythroid differentaition bias				Dong CY, 2014 (90)
SNAI1	Transgenic mouse, Vav-iCre	Increased ST-HSCs, increased GMPs.	Enhanced myelopoiesis, increased immature myeloid cells with enahcned self-renewal and proliferative capacity		Myeloproliferation, AML	Carmichael C, 2020 (96)
	CombiTA-SNAI1 transgenic				AML, B-lymphomas	Perez-Mancera PA, 2005 (97)
SNAI2	CombiTA-SNAI1 transgenic				AML, B-ALL	Perez-Mancera PA, 2005 (97)
SNAI3	Retroviral overexpression and transplant	Normal HSCs from retroviral SNAI3+ cells	Normal myeloid output from retroviral SNAI3+ cells	Reduced lymphoid (B and T) cell output from retroviral SNAI3+ cells		Dahlem T, 2012 (98)

### 3.1 ZEB family

ZEB1 is expressed widely throughout hematopoiesis, with the greatest expression observed in hematopoietic stem and multipotent progenitor cells (HSPCs) as well as in more differentiated myeloid, erythroid, and lymphoid cells. Conversely, ZEB1 expression is significantly lower in committed myeloid-restricted and lymphoid-restricted progenitors (99, 100). *Zeb1* mutant embryos, lacking the C-terminal zinc-finger domain, experience perinatal lethality with ~80% of mice dying within two days of birth (32). Homozygous mutant embryos are morphologically normal; however, they show significant thymic atrophy and drastically reduced thymocyte number, a phenotype that persists in the 20% of mice surviving the perinatal lethality period (32). Thymocyte analysis in surviving mice revealed a significant reduction in both immature and mature T cells, with the majority of detectable thymocytes being double positive (DP) CD4^+^CD8^+^ or single positive (SP) CD4^+^ or CD8^+^ T cells. A concurrent reduction of mature T cells was also observed in the peripheral lymphoid organs of these mice (32). B and myeloid cell development appeared unaffected, with numbers of these cells in the spleen and bone marrow of *Zeb1* mutant mice reported to be normal. A second *Zeb1* mutant mouse with a C-terminal truncation generated through ENU mutagenesis, termed Zeb1*^Cellophane^*, also displayed thymic atrophy and impaired T cell development. The thymus similarly contained a significantly enhanced proportion of immature double negative (DN) T cells and mature SP T cells, alongside a reduced proportion of intermediate DP T cells (85). Despite these thymic abnormalities, the Zeb1*^Cellophane^* mice had normal T cell numbers in the spleen. B cell development was largely normal, although they had a slightly reduced percentage of marginal zone B cells in the spleen and significantly reduced proportion peritoneal B1 cells. These mice also had significantly reduced natural killer (NK) cell numbers, however this phenotype was not described further. Myeloid cell development was not explicitly analyzed in any of these mutant *Zeb1* mouse models.

Almotiri et al. has more recently employed an interferon-inducible Mx1-Cre based approach to conditionally knockout (KO) *Zeb1* in adult hematopoietic cells (100). In this system, two weeks after Mx1-Cre induced *Zeb1* deletion, all KO mice developed reduced monocytic cell numbers but retained normal numbers of granulocytic and lymphoid cells. In line with the constitutive mutant *Zeb1* mice, these inducible *Zeb1* KO mice also displayed reduced thymic cellularity with an increase in the proportion of immature DN T cells and more mature SP T cells, and a concomitant reduction in the proportion of intermediate DP T cells. Within the DN population, a further differentiation block was apparent between the DN1 and DN2/3 stages of maturation. Overall, the authors concluded that the reduced thymocyte cellularity in *Zeb1* conditional KO mice was likely due to enhanced apoptosis in the more mature DP and SP T cells, suggesting *Zeb1* loss impairs thymocyte survival at these later stages of maturation (100). Almotiri et al. also observed reduced CD8^+^ central and effector memory T cells in the blood and bone marrow of their *Zeb1* conditional KO mice. This finding correlates with earlier published data showing ZEB1 expression to be important for the development and maintenance of CD8^+^ T-cell memory (101).

HSCs were present in normal numbers following induction of *Zeb1* KO, however upon competitive transplantation with wild type cells they displayed severe self-renewal and differentiation defects leading to rapid engraftment failure. Bone marrow homing 18 hours post-transplant was normal, demonstrating the migration and invasion capability of *Zeb1* KO HSCs was not impacted. Gene expression analysis of *Zeb1* KO HSCs identified altered expression of EMT related genes, such as those involved in cell adhesion, cell polarity and the cytoskeleton as well as alterations in genes important for both myeloid and lymphoid differentiation (100). In particular, increased expression of the epithelial adhesion molecule EPCAM1 in *Zeb1* KO HSCs was found to enhance their survival by supporting a pro-survival gene expression program, including increased expression of anti-apoptotic BCL-XL, leading to reduced apoptosis. As EPCAM1 is usually downregulated as HSCs differentiate, this increased expression in *Zeb1* KO HSCs also likely contributes to an imbalance between self-renewal and differentiation *in vivo* (100).

Wang et al. independently generated a hematopoietic-restricted KO of *Zeb1* using Tie2-Cre, Vav-iCre or the tamoxifen inducible RosaERT2-cre crossed onto a *Zeb1* floxed background (99). They also generated an inducible *Zeb1/Zeb2* double knockout (DKO) model using the tamoxifen inducible RosaERT2-cre approach. They used these models in combination with bone marrow transplantation studies to examine the role of ZEB1 in hematopoietic differentiation, both alone as well as in collaboration with ZEB2. In these animal models, *Zeb1* KO led to decreased HSPC populations, impaired myeloid cell output (particularly monocytic cells) and reduced thymic cellularity. While absolute numbers were not provided, characterization of T-cell proportions in the thymus revealed a reduced percentage of DN4 T cells and increased percentage of CD8^+^ SP T cells. Differences in the T-cell phenotype described by Wang et al. and Almotiri et al. may reflect the different models utilized, such as the use of bone marrow transplantation models in the Wang et al. study and the potential immune modulating impacts of polyI:polyC treatment in the Mx1-Cre model utilized by Almotiri et al. Nevertheless, *Zeb1* loss clearly impacts T-cell development in the thymus and it will be important for future studies to clarify the role it plays using more sophisticated lineage restricted knockout models.

Wang et al. further demonstrated that *Zeb1* KO HSPCs had impaired self-renewal potential, as evidenced by decreased hematopoietic colony formation in serial replating assays and reduced capacity to give rise to all mature hematopoietic cells in competitive BM repopulation assays. These HSPC defects were more severe in *Zeb1/2* double knockout (DKO) mice, with mice rapidly succumbing to anemia and cytopenia following tamoxifen induced deletion of both genes. Interestingly, a single wildtype allele of *Zeb2* was sufficient to rescue the hematopoietic defects observed in the DKO mice, indicating that ZEB2 might play a more dominant role in regulating hematopoietic lineage differentiation (99).

In other studies, ZEB1 expression has been detected across all dendritic cell (DC) subsets and neutrophils (102) and *in vitro* culture systems have identified a role for this protein in DC activation and subsequent induction of T cell responses (103). Further research, however, is needed to clarify the role/s of ZEB1 in mature myeloid and lymphoid cell subsets.

ZEB2 is also broadly expressed throughout hematopoiesis, with reduced expression in T cells relative to myeloid and B lineage cells (87, 102). A role for ZEB2 during normal hematopoiesis has been studied using a variety of conditional *Zeb2* loss of function mouse models. Hematopoietic-restricted (Vav-Cre) and combined hematopoietic and endothelial-restricted (Tie2-Cre) KO of *Zeb2* was utilized by Goossens et al. to study the role of ZEB2 in HSC formation and differentiation during embryonic hematopoiesis (87). While ZEB2 was not required for HSC cluster formation in the embryonic AGM region, it played a crucial role in HSPC differentiation and migration. *Zeb2* KO embryos displayed a severe block in hematopoietic differentiation in all lineages, as evidenced by reduced development of mature blood cells *in vivo* and impaired differentiation in *in vitro* methylcellulose cultures. In addition, *Zeb2* KO embryos showed significant alterations in the localization of HSPCs in the fetal liver, a significant reduction in circulating HSPCs as well as decreased homing of hematopoietic cells to the bone marrow compared with wildtype controls (87). This was attributed to an aberrant increase in the expression of β1 integrin and CXCR4, previously shown to be crucial for HSC mobilization and homing (104–106). Moreover, *Zeb2* KO fetal livers contained increased numbers of HSCs, pointing toward a possible feedback loop compensating for the hematopoietic differentiation block and/or enhanced retention of HSPCs. Interestingly, *Zeb2* deficiency also resulted in high embryonic/neonatal lethality due to intracephalic hemorrhaging. This was proposed to be due to significantly reduced angiopoietin-1 expression and subsequently impaired pericyte coverage of vasculature (87). A similar lethality was not observed in *Zeb1* deficiency models described earlier that were generated using the same approach by Wang et al. (86).

Li et al. generated conditional *Zeb2* KO in adult hematopoietic cells using the interferon-inducible Mx1-Cre approach. *Zeb2* deletion using this model resulted in an increased frequency of HSPCs in the BM and an expansion of megakaryocyte-erythroid progenitors (MEPs) with concomitant reduction of granulocyte-monocyte progenitors (GMPs). Bone marrow in these mice also displayed a reduction in B cells (due to a block in transition from pre-pro-B to pro-B), monocytes and mature erythroid cells along with a significant expansion of granulocytes and immature megakaryocytes (88). The mice also developed splenomegaly, extramedullary hematopoiesis and bone marrow fibrosis suggestive of a myeloproliferative phenotype. Bone marrow transplantation assays provided evidence that *Zeb2* KO did not alter HSC self-renewal but confirmed their impaired differentiation capacity. These assays also demonstrated that hematopoietic abnormalities in *Zeb2* KO mice were not a consequence of an impaired BM niche (88). Mechanistically, Li et al. identified impaired responsiveness of ZEB2 KO bone marrow cells to IL-3 and IL-6 cytokine signaling but enhanced responsiveness to G-CSF stimulation. This latter finding likely contributing to the predominant granulopoiesis observed in ZEB2 KO mice.

**Figure 2:**
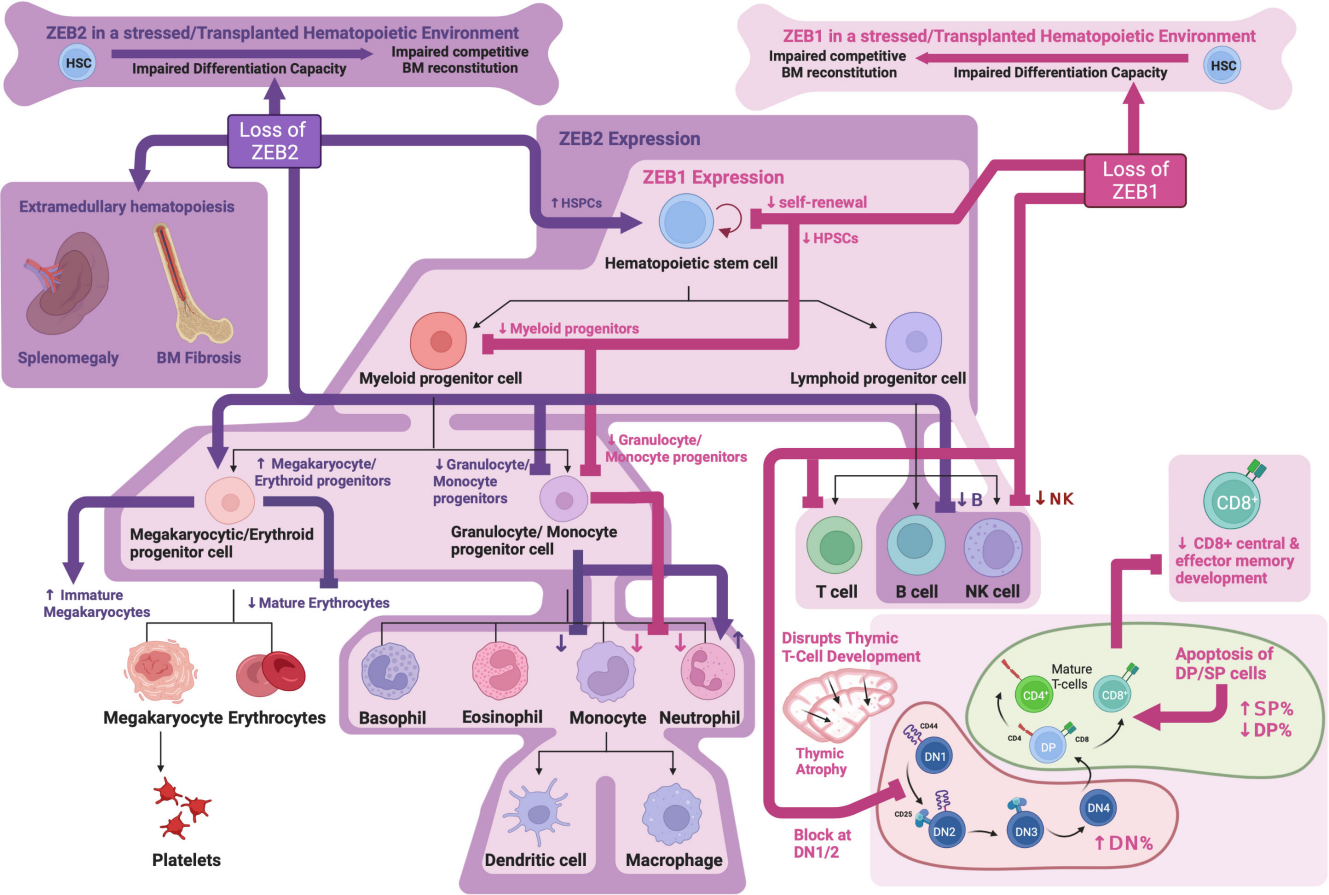
ZEB family roles in hematopoiesis: Schematic showing known functions of ZEB1 and ZEB2 during normal hematopoiesis as determined through analysis of knockout mouse models. Created with BioRender.com.

Studies looking at *Zeb2* KO or overexpression during DC development have demonstrated that ZEB2 is required for the development of a subset of DCs and is thought to play a role in maintaining their cell fate or identity (107–109). Mechanistically, Zeb2 was shown to directly repress expression of *Id2*, which negatively impacts plasmacytoid DC (pDC) development. These data implicate *Id2* repression as a mechanism by which ZEB2 drives pDC development (108). Similar roles for ZEB2 in maintaining monocytic (109) and tissue-resident macrophage cell identity have also been identified (110) although the key mechanisms involved remain to be elucidated. While *Zeb2* KO mice do not display overt T cell abnormalities, ZEB2 has been shown to be upregulated following CD8^+^ T cell activation and is important for promoting CD8^+^ T effector cell differentiation and survival (111, 112). Interestingly, again here ZEB2’s role in CD8^+^ T effector cell regulation has been contributed, at least partially, to *Id2* repression which is important for CD8^+^ effector memory differentiation (113, 114). See **Figure 2** for an overview of ZEB family roles in hematopoiesis.

### 3.2 TWIST family

TWIST1 is highly expressed in embryonic HSPCs in the AGM region at E9.5 and E10.5 with significantly lower expression in HSPCs in the E14.5 fetal liver (89, 115). *Twist1* KO is embryonically lethal due to vascular and cranial neural tube defects around E11.5 (10). Kulkeaw et al. found that while *Twist1* deficiency did not affect formation of embryonic HSPCs themselves, it instead impaired embryonic HSPC differentiation (89). This was evidenced by reduced numbers of myeloid and erythroid colonies in *in vitro* colony assays using *Twist1* KO E10.5 AGM-derived cells, as well as impaired B lymphoid differentiation following culture on an OP9 cell layer. Mechanistically, TWIST1 controls embryonic HSPC differentiation, at least partially, through direct regulation of MYB and GATA2 expression (89).

In the adult hematopoietic compartment, TWIST1 expression is most abundant in long-term HSCs (LT-HSCs) and short-term HSCs (ST-HSCs), with its expression declining during differentiation (90, 91). Enforced expression of TWIST1 in HSCs enhanced their ability to repopulate the bone marrow long term following competitive transplantation alongside wild type HSCs, while loss of TWIST1 led to a reduced ability of HSCs to engraft in a similar experiment. TWIST1 overexpressing HSCs also displayed enhanced quiescence and increased self-renewal potential, as well as a specific myeloid/erythroid differentiation bias. These phenotypes were associated with activation of the myeloid lineage-determining factors PU.1 and GATA-1 and downregulation of the lymphoid factor GATA-3 and HSC regulator RUNX1 (90). Conditional *Twist1* KO using an Mx1-Cre based approach in the adult hematopoietic system resulted in reduced HSC numbers with impaired quiescence and self-renewal capacity. Furthermore, *Twist1* KO HSCs had reduced lymphoid and megakaryocyte/erythroid differentiation ability with a concomitant increase in granulocyte/macrophage differentiation capacity (91). *Twist1* KO HSCs also had significantly reduced engraftment capacity in a competitive bone marrow transplant setting, which was not due to any observable homing defect. The impact of *Twist1* deletion during stress hematopoiesis was examined following irradiation or treatment with the chemotherapeutic drug, 5-Fluorouracil (5-FU). This analysis revealed an important role for TWIST1 in protecting HSCs from irradiation-induced apoptosis, senescence and DNA damage. Treatment with 5-FU also led to significantly reduced bone marrow cellularity and impaired HSC recovery in *Twist1* KO mice after a single dose, and rapid HSC exhaustion and mouse death following serial 5-FU treatments. Mechanistically, *Twist1* KO resulted in enhanced mitochondrial calcium levels and subsequently increased production of reactive oxygen species (ROS) in lymphoid-biased HSCs but not myeloid-biased HSCs following irradiation induced stress. Importantly, blockage of voltage-gated calcium channels was largely able to reverse irradiation induced death in *Twist1* KO mice as well as rescue HSC levels, demonstrating a key role for enhanced mitochondrial calcium influx in driving the stress induced hematopoietic phenotype in these mice (91).

TWIST1 is also known to play a role in mesenchymal stem cell (MSC) proliferation, survival and differentiation (116–119). Interestingly, *Twist1* KO in the bone marrow niche compartment (including MSCs) resulted in reduced homing of wild type HSCs following irradiation and transplantation. Wild type HSCs in a *Twist1* deficient bone marrow microenvironment also displayed reduced quiescence and self-renewal potential with enhanced proliferation and a clear myeloid lineage bias. There was also reduced retention of wild type HSCs in *Twist1* deficient bone marrow, with enhanced mobilization to the spleen and blood - likely due to an observed reduction in expression of CXCL12 and VCAM1 (118). Interestingly, increased TWIST1 expression in bone marrow-derived mesenchymal stem/stromal cells (BMSC) enhanced their ability to maintain CD34^+^ hematopoietic stem cells (HSC) in long-term *in vitro* cultures (116). This was likely mediated, at least partially, by direct activation of the *Cxcl12* gene by TWIST1. These findings demonstrate a clear role for TWIST1 expression in bone marrow niche support of HSCs likely through regulation of CXCL12 expression, a protein known to be important for supporting HSC survival and self-renewal and also involved in protecting HSCs from oxidative stress (120, 121)

TWIST2 is also expressed in the hematopoietic compartment, preferentially in myeloid progenitors (92), where it plays a key role in suppressing myeloid differentiation. *Twist2* silencing in embryonic stem cells leads to enhanced generation of myeloid lineage cells during *in vitro* hematopoietic differentiation (122), while *Twist2* deficient mice show significantly increased numbers of immature and mature myeloid cells across all hematopoietic organs, including macrophages, neutrophils and basophils (92). The significant basophilia as well as the presence of hyper-segmented neutrophils and atypical monocytes were suggestive of a myelodysplastic/myeloproliferative phenotype. No significant alteration in the numbers of lymphocytes, red blood cells or platelets was observed in these mice. The increase in total myeloid cells likely resulted from an overall expansion of myeloid progenitors in the bone marrow of *Twist2* KO mice, particularly the granulocyte/macrophage progenitor (GMP) which showed increased proliferation and differentiation capability in *in vitro* assays. The myeloid skewed and enhanced differentiation of *Twist2* KO progenitors was also observed in both non-competitive and competitive bone marrow transplant experiments, demonstrating a cell-intrinsic effect of *Twist2* KO as well as a strong competitive advantage against wild type cells. Mechanistically, TWIST2 was found to inhibit the activity of known regulators of myeloid differentiation, RUNX1 and C/EBPα, as well as suppress the production of pro-inflammatory cytokines and chemokines (92).

Interestingly, *Twist2* KO mice also develop an inflammatory syndrome shortly after birth due to enhanced pro-inflammatory cytokine production that results in perinatal death within 3-4 weeks after birth (123). A possible role for TWIST2 in the regulation of inflammation is further supported by its high expression in chronically activated T helper (Th) lymphocytes (124), and ability to repress the expression of key pro-inflammatory cytokines such as TNFα, IL1β and IFNγ (123, 125–128).

**Figure 3:**
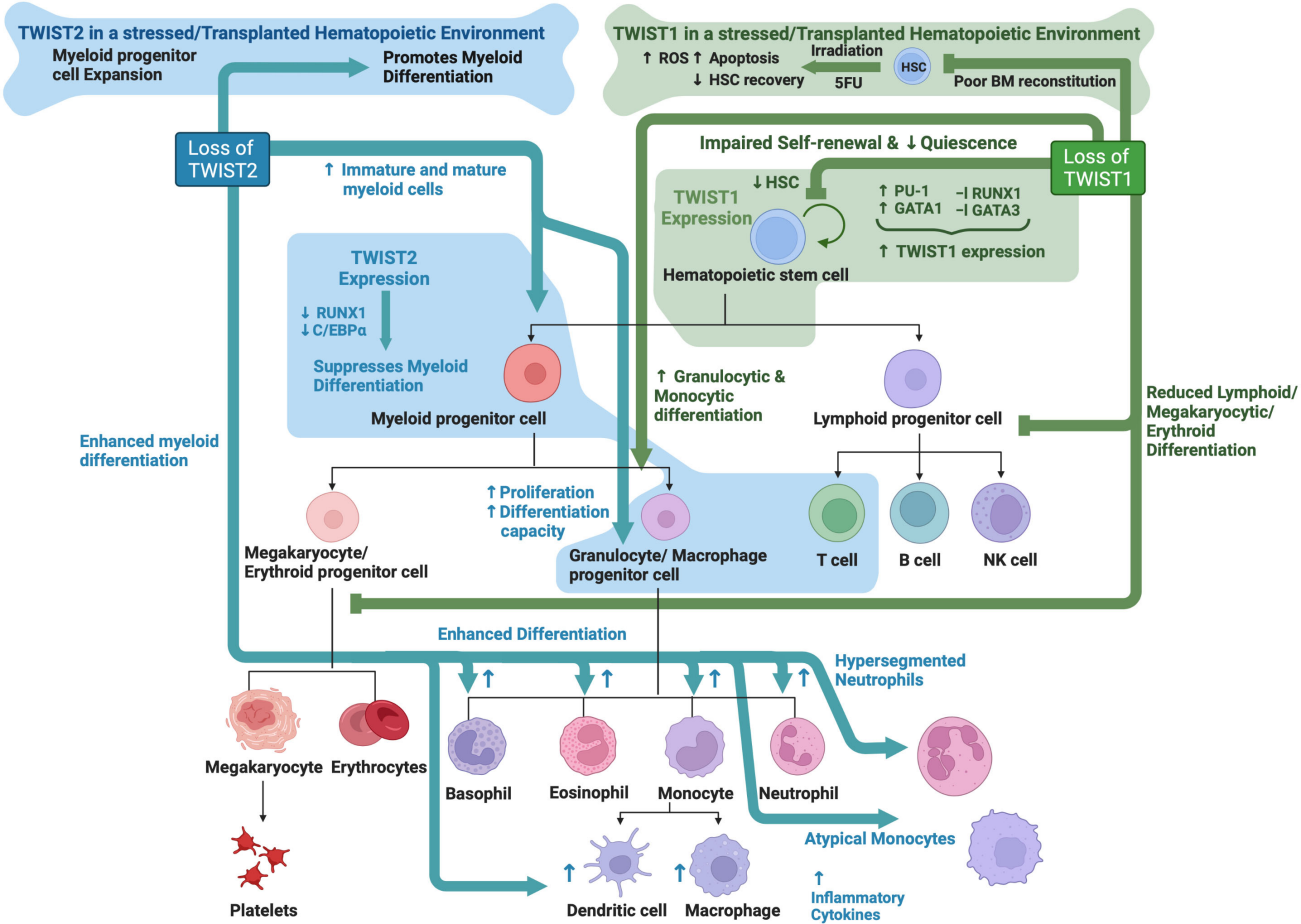
TWIST family roles in hematopoiesis: Schematic showing known functions of TWIST1 and TWIST2 during normal hematopoiesis as determined through analysis of knockout mouse models. Created with BioRender.com.

While no obvious T or B lymphoid phenotype was identified in *Twist2* KO mice, TWIST2 has been documented to play a role in regulating T cell selection and apoptosis in the developing thymus (129–131). Furthermore, Hwang et al. found that TWIST2 expression is important for regulating the CD4/CD8 thymocyte lineage determination downstream of TCR activation (132). See **Figure 3** for an overview TWIST family roles in hematopoiesis.

### 3.3 SNAIL family

In the hematopoietic compartment SNAI1 and SNAI3 are expressed in mature T and B cells, with SNAI1 also expressed in mature myeloid lineage cells (76, 77), whereas SNAI2 has only been detected in hematopoietic stem and progenitor cells (77, 94). *Snai1* KO is embryonically lethal at E7.5-8.5, thus precluding studies being undertaken into the role of SNAI1 during hematopoiesis (65). A hematopoietic specific *Snai1* KO showed no overt phenotype, suggesting that SNAI1 is not required for normal hematopoiesis, or alternatively that other family members may be able to compensate for SNAI1 loss (93). A deeper investigation of this mouse model, however, is still required. Hematopoietic specific *Snai1* transgenic mice, on the other hand, develop a myeloproliferative phenotype characterized by an expanded population of both immature and mature myeloid cells (particularly granulocytes), disrupted bone marrow and spleen architecture and evidence of extramedullary hematopoiesis. Interestingly, some of these mice developed Acute Myeloid Leukemia (AML), which will be discussed more later.

*Snai2* KO mice display normal B and myeloid cell development, however they show macrocytic anemia as well as abnormal T cell development characterized by reduced thymus size and reduced numbers of CD4^+^CD8^+^ DP T cells (72). The reduced thymus size and thymocyte numbers correlated with increased T cell apoptosis as demonstrated by an increase in apoptotic bodies and TUNEL positive cells in histological sections. In addition to the macrocytic anemia observed at steady state, stress erythropoiesis was also perturbed in *Snai2* KO mice as demonstrated by reduced erythroid recovery following *in vivo* hematopoietic stress driven by either phenylhydrazine (PHZ)-induced hemolytic anemia or pregnancy-induced anemia. This impaired stress erythropoietic response was likely due to reduced numbers of BFU-E and CFU-E in the spleen of *Snai2* KO mice at steady state, and a significant reduction in their ability to expand under stress conditions (72). A role for SNAI2 downstream of SCF/cKIT signaling in HSCs was postulated based on similar phenotypes observed between *Snai2* KO mice and *cKit* or *Scf* mutant mice, supported by data showing induction of *Snai2* expression upon *Scf* stimulation of *cKit in vitro* and anemia-induced activation of *cKit* signaling *in vivo* (72). In a follow up study these authors further found that, similarly to *cKit* or *Scf* mutant mice, *Snai2* KO bone marrow cells were also significantly radio-sensitive. Impaired hematopoietic recovery following low dose irradiation resulted in death in the majority of *Snai2* KO mice as compared to 100% survival in wild type controls. Importantly, intraperitoneal injection of a TAT-SNAI2 fusion protein that readily enters cells was able to rescue irradiation induced death not only in *Snai2* null mice but also in *cKit* mutant mice demonstrating a key role downstream of cKit/SCF signaling in radioprotection of HSCs (133).

A separate study by Inoue et al. performed an extensive analysis of the hematopoietic system of an independently generated *Snai2* KO mouse model (94). *Snai2* KO mice had normal peripheral blood cell counts, however the number of *in vitro* colony-forming progenitors (BFU-E, CFU-E, CFU-GM, and CFU) was slightly increased relative to wild type mice. In contrast to their relatively normal steady state hematopoietic development, *Snai2* KO mice were severely impaired in their ability to reconstitute their bone marrow following total body irradiation (TBI). *Snai2* KO mice showed increased sensitivity to DNA damage induced by irradiation and all *Snai2* KO mice died by day 13 post irradiation due to severe pancytopenia. By comparison, wild type and *Snai2* heterozygous mice survived longer, with around 50% surviving to at least 30 days post irradiation. In response to irradiation, *Snai2* KO HSPCs displayed significantly increased apoptosis as compared with wild-type HSPCs, suggesting a role for SNAI2 in protecting against DNA damage induced cell death (94). In a follow up study, Wu et al. found that wild type mice previously reconstituted with *Snai2* KO bone marrow were just as sensitive to irradiation induced death as *Snai2 KO* mice, demonstrating that the increased sensitivity of *Snai2* KO HSPCs to irradiation was cell intrinsic. Importantly, the authors also discovered that this radio-sensitivity of *Snai2* KO HSPCs could be rescued by transgenic expression of the antiapoptotic protein BCL2 or deletion of TP53. Further, it was demonstrated that SNAI2 is upregulated by TP53 following irradiation, and in turn it can transcriptionally repress the BH3-only pro-apoptotic protein, PUMA leading to an antagonism of TP53 induced apoptosis. These data indicate that SNAI2 plays a key role in mediating the DNA damage response downstream of the TP53 pathway in HSPCs (134).

In a third study, Sun et al. further examined the functional capacity of *Snai2* KO HSCs (95). Using competitive bone marrow transplantation experiments these authors demonstrated that *Snai2* KO HSCs had increased proliferative capacity and enhanced ability for hematopoietic reconstitution, with an approximately 8-fold higher repopulation efficiency as compared to *Snai2* heterozygous HSCs. Importantly, this enhanced reconstitution ability was not due to an altered differentiation or homing capacity. *Snai2* KO HSCs also displayed increased self-renewal capacity as demonstrated by limiting dilution and serial transplantation experiments. Following treatment with the chemotherapeutic drug 5-fluorouracil (5FU), which kills proliferating cells and drives quiescent HSCs into cell cycle, *Snai2* KO HSCs showed enhanced proliferation and expansion compared to WT cells both *in vitro* and *in vivo*. This enhanced HSC proliferation and expansion of *Snai2* KO HSCs following 5FU treatment also lead to superior repopulating ability upon competitive transplantation with wild type cells into irradiated recipient mice (95). The percentage of *Snai2* KO HSCs in S phase was also significantly higher than for wild type HSCs, supporting the idea that quiescent *Snai2* KO HSCs were induced into cell cycle more effectively by 5FU than wild type HSCs. No difference in the level of 5FU-induced apoptosis was observed in *Snai2* KO HSCs. Together, these data suggest that SNAI2 acts as a negative regulator of HSC self-renewal and proliferation, and a positive regulator of HSC quiescence.

While the above studies suggested that the hematopoietic defects in *Snai2* KO mice were hematopoietic cell intrinsic, Wei et al. identified a potential extrinsic role for SNAI2 in the bone marrow niche (135). Following exposure to a lethal dose of irradiation (12Gy), *Snai2* KO mice could not be rescued from irradiation-induced death *via* transplantation of wild type bone marrow cells, with the majority of mice dying by three weeks post irradiation and transplantation. In contrast, 100% of wild type mice receiving either wild type bone marrow or *Snai2* KO bone marrow survived. These findings are somewhat contradictory to those of Wu et al. who previously found that *Snai2* KO mice could in fact be rescued from irradiation-induced death by transplantation of wild type bone marrow cells (134). This discrepancy may be explained by the use of a lower dose of irradiation by Wu et al. (7Gy) or different genetic backgrounds of the *Snai2* KO mice between the two studies. Interestingly, Wu et al. had also demonstrated that following complete bone marrow reconstitution, a second dose of irradiation (7Gy) still induced bone marrow failure and death in wild type mice with *Snai2* KO bone marrow, whereas *Snai2* KO mice with wild type bone marrow were protected (134). Combined these data suggest that extrinsic SNAI2 in the bone marrow niche is crucial for enabling HSPC engraftment and hematopoietic reconstitution following irradiation, whereas intrinsic SNAI2 expression in the HPSC compartment is important for protecting against irradiation-induced cell death.

The first evidence of a role for SNAI3 in the hematopoietic system came from a study that examined the negative regulatory element of the mouse *Itgb2l*, which is preferentially expressed in maturing neutrophils (136). Using an electrophoretic mobility shift assay (EMSA) it was demonstrated that SNAI3 could bind to the negative regulatory element on the *Itgb2l* gene and block the transcriptional activator, PU.1, from binding and driving transcription. Another study by Dhalem et al. examined hematopoietic lineage differentiation and derivation of mature hematopoietic cells upon retroviral mediated over-expression of SNAI3 in mouse HSPCs (98). Mice transplanted with SNAI3 expressing HSPCs (marked by GFP expression) had an almost complete loss of GFP^+^ T and B lymphoid cells, with the GFP^+^ cells being primarily myeloid. Interestingly, the GFP^+^ HSPC compartment appeared relatively normal in these mice as compared to control mice receiving HSCPs transduced with an empty vector control retrovirus (98). These data demonstrate that aberrant expression of SNAI3 significantly perturbs lymphoid differentiation but has minimal if any impact on early HSPC development and myeloid differentiation.

**Figure 4:**
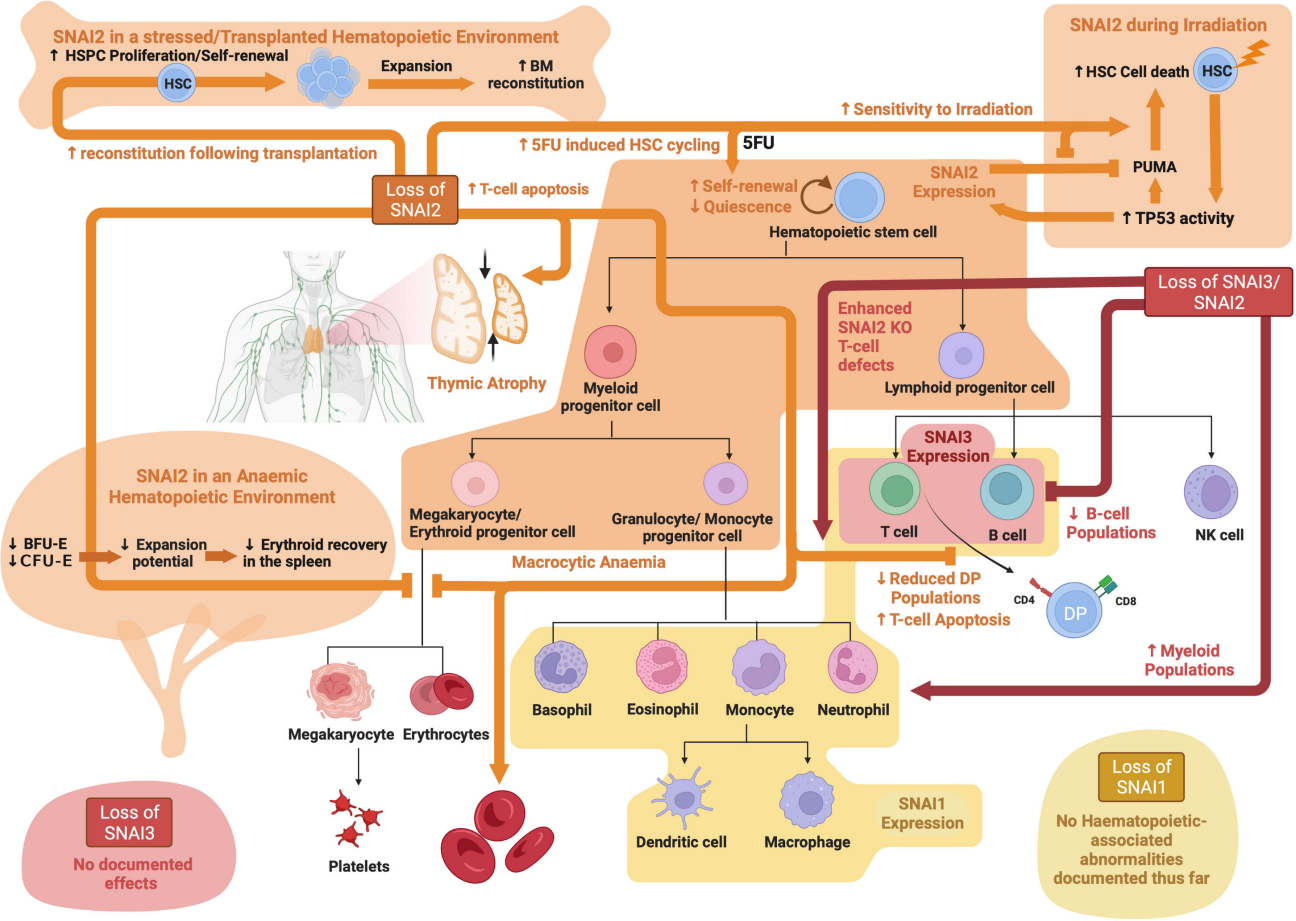
SNAIL family roles in hematopoiesis: Schematic showing known functions of SNAI1, SNAI2 and SNAI3 during normal hematopoiesis as determined through analysis of knockout mouse models. Created with BioRender.com.

*Snai3* KO mice are completely viable with no obvious phenotypic defects, demonstrating that SNAI3 is not essential for embryogenesis or steady state adult development (76). Pioli et al. generated a conditional Cre-mediated *Snai3* knockout mouse model and performed a T cell specific *Snai3* deletion using Lck-Cre. Deletion of *Snai3* in the T cell lineage had no effect on T cell development in the thymus and no T cell abnormalities were observed in the peripheral lymphoid organs. To test for possible functional redundancy between SNAI2 and SNAI3 in T cells, Pioli et al. further generated *Snai2/Snai3* double knockout (DKO) mice. These DKO mice had a more severe phenotype as compared to either single KO mouse, with severe growth retardation, infertility and almost complete lethality by 15 weeks of age (77). Analysis of lymphoid organs revealed that DKO mice had a significantly reduced thymus size (even when normalized for the reduced body weight), a decreased proportion of DP (CD4^+^CD8^+^) thymocytes with a concomitant increase in CD4^+^ and CD8^+^ SP cells. Surprisingly, the distribution of CD4^+^ and CD8^+^ SP T cells in peripheral organs was relatively normal. DKO mice also displayed significantly reduced B cell numbers and increased myeloid cells in the marrow, spleen and blood (77). Whether the increase in myeloid cells was a direct result of *Snai2* and *Snai3* loss in these cells or was rather due to the striking loss of B cells still remains to be elucidated. No analysis was performed on the primitive HSPC compartment of these mice and so it is not known what impact combined loss of *Snai2* and *Snai3* might have at earlier stages of hematopoietic development (77). This study however did clearly indicate potentially redundant functions for SNAI2 and SNAI3 during later stages of hematopoiesis. Similar studies using *Snai1* knockout in combination with either *Snai2* and/or *Snai3* would provide important additional knowledge in this area. See **Figure 4** for an overview of SNAIL family roles in hematopoiesis.

## 4 EMT transcription factors in hematological malignancy

While there is still much to be learned regarding the exact mechanisms involved, it is becoming increasingly evident that EMT-TFs are important regulators of normal blood cell development and function. It is perhaps not surprising, therefore, that aberrant expression and/or function of EMT-TFs is also now emerging as a novel and important contributor to the malignant hematopoietic phenotype.

## 5 Myeloid malignancies

### 5.1 ZEB family

Using publicly available RNA-sequencing data from the GEPIA database (http://gepia.cancer-pku.cn), Li et al. identified high *ZEB1* expression in AML patients and found it to be associated with a worse overall survival (137). A similar association between high ZEB1 expression and worse overall survival had also been shown by Stavropoulou et al. in their own AML patient cohort, and indeed ZEB1 expression was significantly higher in AMLs with a more stem-cell like and aggressive phenotype (138). Shousha et al. identified a 1.8 fold increase in *ZEB1* mRNA expression in more than half of their AML patients as compared to control subjects, using qRT-PCR analysis on peripheral blood samples (139). In contrast to the above studies, Almotiri et al. used publicly available Affymetrix microarray data to describe *ZEB1* expression as being lower in AML patient samples compared to normal cells (100). The use of datasets generated using alternative gene expression analysis technologies may explain the discrepant results between these studies, however additional investigation is warranted to clarify whether aberrant *ZEB1* expression is indeed a significant finding in AML.

ZEB1 appears to play important roles in AML cell biology, with siRNA mediated knockdown of *ZEB1* in human AML cell lines leading to reduced cell proliferation and induced myeloid cell marker expression *in vitro*, and subsequently delayed tumor onset in *in vivo* xenograft models (137). Extending these studies to primary mouse AML models, Stavropoulou et al. demonstrated that shRNA mediated knockdown of *Zeb1* in an MLL-AF9 driven AML model resulted in impaired tumor cell invasion *in vitro* and reduced *in vivo* infiltration into the bone marrow 1-week post-transplant (138). In contrast, Almotiri et al. found that Cre-mediated knockout of *Zeb1* in either a MLL-AF9 or Meisa1/Hoxa9 mouse model of AML actually enhanced tumor development *in vivo* (100). These stark differences may be due to the use of distinct models of *Zeb1* perturbation, with Stavropolou et al. and Li et al. using a stable shRNA knockdown approach, where the cells already had reduced ZEB1 expression prior to transplant, and Almotri et al. using an Mx1-Cre model to induce *Zeb1* knockout after AML was established *in vivo*. It is also important to note that Stavropoulou et al. did not extend their animal studies to study tumor development post 1-week and thus no data on disease progression and latency is available. These data do, however, pose the question as to whether ZEB1 may play opposing roles in driving tumor cell engraftment on one hand, while impairing tumor cell proliferation on the other. This would not, however, agree with the observed negative impact of *ZEB1* knockdown on cell proliferation in AML cell lines *in vitro.* Stavropoulou et al. further determined that high ZEB1 expression was particularly associated with a more immature and stem cell like AML phenotype generated by transducing the MLL-AF9 oncogene virally into long term repopulating HSCs (LT-HSCs) as opposed to more differentiated granulocyte/macrophage progenitors (GMPs). These HSC-derived AMLs were also more invasive with higher numbers of leukemia initiating cells (LICs) *in vivo* (138).

Mechanistically, Li et al. found ZEB1 expression in AML to be linked to altered TP53 protein levels, with knockdown of *Zeb1* leading to enhanced TP53 protein levels and overexpression resulting in reduced TP53 protein levels (137). Whether this is due to direct effects on TP53 transcription, translation or protein stability remains to be determined. The authors further suggested that this ZEB1 mediated regulation of TP53 may occur *via* the PTEN/PI3K/AKT signaling pathway, but again clear mechanistic insight remains to be elucidated.

Expression of *ZEB2* does not appear to be specifically increased in AML cells, with its expression level in AML being similar to that of normal HSPCs. Similarly, no correlation has yet been demonstrated between *ZEB2* expression and survival in AML. Despite ZEB2 not being specifically upregulated in AML cells, its expression was found to be significantly increased following transduction of the *AML-ETO* oncogene into a mouse hematopoietic progenitor cell line. Furthermore, high ZEB2 expression was specifically associated with an invasive phenotype and EMT-like gene expression signature in these cells (140). In human AML cell lines, Li et al. were able to show that shRNA mediated knockdown or CRISPR mediated knockout of ZEB2 reduced cell growth and induced aberrant myeloid differentiation *in vitro* (141). Furthermore, shRNA knockdown of *Zeb2* in mouse MLL-AF9 AML cells led to reduced leukemia cell proliferation *in vitro* (141). A similar finding was obtained by Wang et al. using a RosaERT2Cre-mediated knock out of *Zeb2* in the MLL-AF9 driven mouse AML model (86). Interestingly, when the authors introduced a double knockout of *Zeb2* and *Zeb1* in this same MLL-AF9 model they did not observe any further delay in tumor onset suggesting that *Zeb1* loss was could not compound the effect of *Zeb2* loss alone.

**Figure 5:**
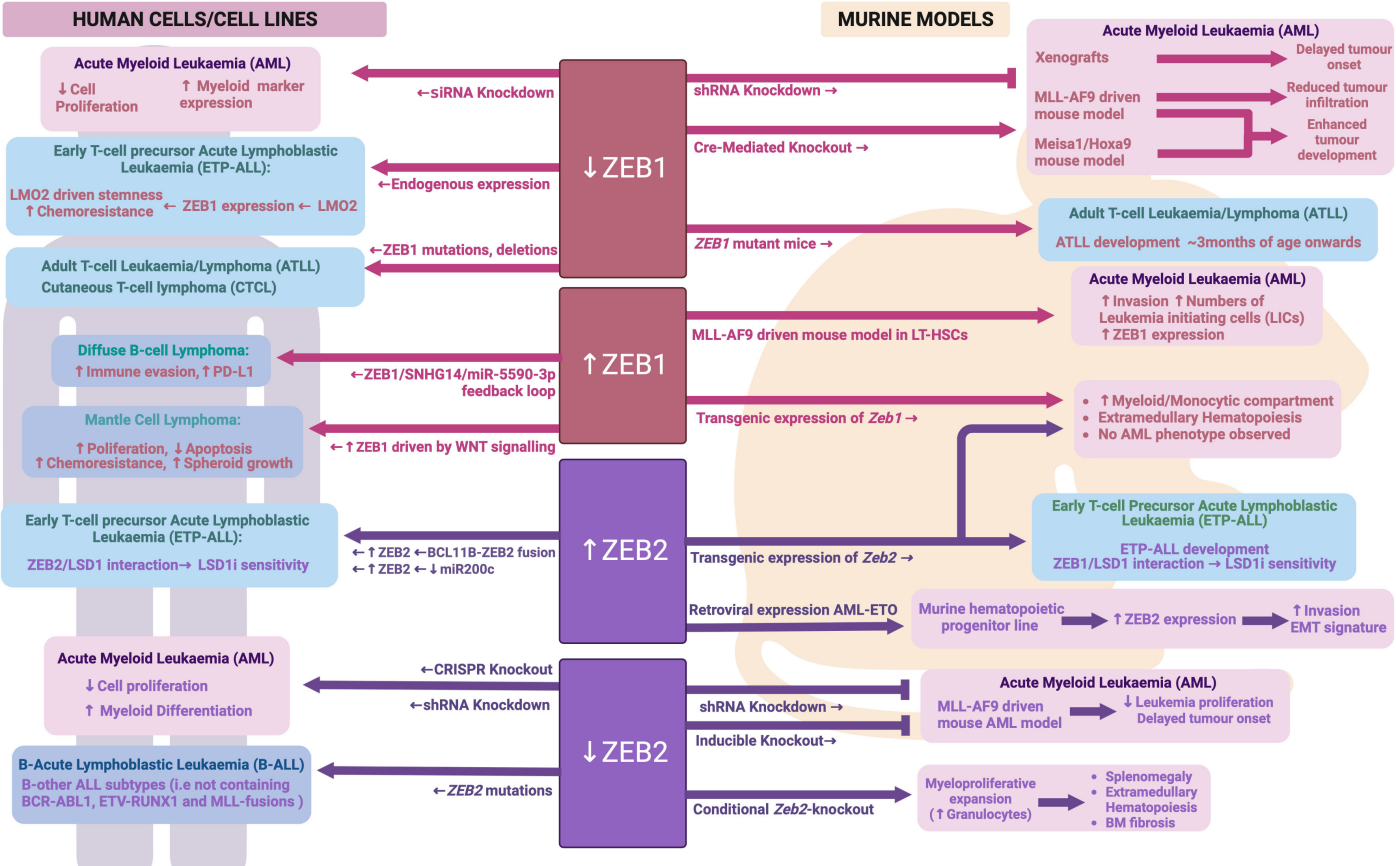
ZEB family during malignant hematopoiesis: Schematic outlining ZEB family roles in malignant hematopoiesis as determined through human and mouse model analyses. Created with BioRender.com.

Strikingly, hematopoietic specific expression of either a *Zeb1* or *Zeb2* transgene in mice led to a significantly expanded myeloid compartment (predominantly monocytic) and development of extramedullary hematopoiesis (86). No AML was observed in these mice up to 1.5 years of age suggesting that while these genes may contribute to AML pathogenesis, they are not strong drivers of AML and likely act in concert with other AML mutations or oncogenes. Somewhat surprisingly, loss of *Zeb2* during adult hematopoiesis was also found to drive development of a myeloproliferative-like phenotype characterized by splenomegaly, extramedullary hematopoiesis and bone marrow fibrosis (88). In contrast to *Zeb2* transgenic mice, where enhanced myeloid development favored the monocytic lineage, these *Zeb2* knockout mice showed enhanced granulocyte production. Mechanistically, Li et al. identified deficient JAK/STAT signaling responses in *Zeb2* KO bone marrow cells when stimulated with IL6 or IL3, but enhanced signaling when stimulated with the granulocyte cytokine G-CSF (88). Furthermore, Pellman and colleagues determined that ZEB2 expression in AML regulates genes important for granulocytic differentiation, likely through interaction with key epigenetic proteins such as LSD1 and HDACs (141). Combined, these data suggest that correct dosage of ZEB transcription factors is important for normal myeloid development and their expression levels may impact different lineages variably – possibly through regulation of key lineage specific cytokine signaling pathways and gene expression networks. See **Figure 5** for an overview of ZEB family in malignant hematopoiesis.

### 5.2 Twist family

TWIST1 expression is highly upregulated in malignant HSCs from Myelodysplastic syndrome (MDS) patients, with its expression increasing with more advanced disease (142). In contrast, there appears to be reciprocally lower levels of TWIST1 expression in the surrounding bone marrow mesenchymal cells in MDS patients, reducing with disease severity. Li et al. further found that levels of TWIST1 in MDS cells resulted in enhanced resistance to TNFα driven apoptosis, TNFα being a pro-inflammatory cytokine that is highly expressed in the MDS bone marrow microenvironment. Knockdown of *TWIST1* in MDS cell lines rendered them more sensitive to TNFα induced cell death, with this thought to be at least in part driven by coordinated regulation of apoptosis by TWIST1, miRs10a/b, NFkB and TP53 (142, 143).

A more recent study by this same group found that TWIST1 expression was actually higher in MDS patients that were non-responsive to treatment with the DNA demethylating agent 5-aza-2’-deoxycytidine, compared to those that were responsive (144). The level of responsiveness was also correlated with increased DNA methylation and expression of the *de novo* DNA methyltransferase, DNMT3A. A direct interaction between TWIST1 and DNMT3A was identified, with evidence provided to suggest this complex can methylate and repress expression of the cyclin dependent kinase inhibitors CDKN1A and CDKN1C. As 5-aza-2’-deoxycytidine treatment induces cell cycle arrest in MDS cells, TWIST1 driven loss of cell cycle inhibition and reduced G0/G1 arrest may contribute to an enhanced resistance to 5-aza-2’-deoxycytidine treatment upon TWIST1 expression. Furthermore, augmented *de novo* DNA methylation through increased DNMT3A levels in TWIST1 expressing cells likely also contributes to reduced sensitivity to the demethylating activity of 5-aza-2’-deoxycytidine.

TWIST1 expression is also upregulated in AML samples, however the impact of this expression on prognosis in AML remains somewhat controversial. One study has found that patients with high TWIST1 expression were more likely to achieve remission following standard AML induction chemotherapy (cytarabine and daunorubicin combination therapy) than those with lower TWIST1 expression, and subsequently achieved a greater overall survival (145). The authors further found that enforced TWIST1 expression in a single AML cell line (KG1a) led to enhanced sensitivity to cytarabine but no change in response to daunorubicin. In contrast, a second study determined that enforced TWIST1 overexpression in two independent AML cell lines (U937 and K562) led to increased resistance to daunorubicin, mitoxantrone or imatinib, and subsequently found that high TWIST1 in AML samples was associated with a worse overall survival (146). The reason for these discordant findings remains unclear, however it may relate, at least partially, to the different ways of stratifying AML patients for survival analysis. For example, Chen et al. included only patients that had received standard of care chemotherapy, while Wang et al. included all AML patients in their analysis.

Wang et al. went on to further show that TWIST1 was most highly expressed in the putative leukemia stem cell (LSC) compartment in AML (CD34^+^CD38^-^) and that its expression in LSCs was higher than in normal CD34^+^CD38^-^ HSCs. They also found that enforced TWIST1 expression could drive increased cell proliferation and enhanced colony formation along with reduced apoptosis in AML cell lines. *TWIST1* knockdown, on the other hand, led to reduced cell proliferation and colony formation and increased apoptosis (146). *TWIST1* knockdown in the K562 AML cell line delayed AML onset in *in vivo* xenograft experiments, while knockdown in LSCs isolated from AML patients led to significantly reduced colony forming potential *in vitro*. These data implicate TWIST1 in regulation of LSC function, which mechanistically may relate to the direct regulation of BMI1 expression, a critical regulator of HSC self-renewal, and indirect regulation of RUNX1 and MPL expression, both important modulators of HSC function and proliferation (146).

TWIST1 expression has been particularly associated with the M3 subtype of AML, also termed Acute Promyelocytic Leukemia (APL), which is driven by the t(15;17) translocation (146, 147). Knockdown of *TWIST1* in the NB4 APL cell line or in a mouse model of APL resulted in apoptosis and differentiation of AML blasts *in vitro* and enhanced survival of transplanted mice *in vivo* (147). In other non-APL subtypes of AML, an association between TWIST1 expression and DNMT3A mutation (a key driver mutation identified in around a third of AMLs) has also been identified, with TWIST1 expression being higher in AML cells carrying mutant DNMT3A (148). Furthermore, mutant DNMT3A but not wild type was able to upregulate TWIST1 when ectopically expressed in an AML cell line. Knockdown of TWIST1 in a DNMT3A mutant AML cell line (OCI-AML3) led to reduced invasion of these cells into the central nervous system of xenografted mice.

While TWIST1 appears to have a clear tumor promoting role in AML cells, its expression in the bone marrow microenvironment seems to have a more tumor inhibiting impact on AML cells. Liu et al. found that deletion of *Twist1* specifically in the bone marrow microenvironment resulted in enhanced engraftment and increased dissemination/infiltration of wild-type murine MLL-AF9 leukemia cells (118). Mechanistically, activated Notch signaling was observed within the *Twist1* deleted niche, which has been shown to contribute to enhanced LSC expansion and self-renewal.

In Chronic Myeloid Leukemia (CML), TWIST1 expression is also upregulated compared to normal samples, with expression increasing further during more advanced phases of the disease (146, 149). More than 90% of CML cases are driven by the BCR-ABL fusion, which is uniquely sensitive to tyrosine kinase inhibitors (TKI) such as imatinib. In samples from CML patients that did not respond to TKI treatment, TWIST expression was 100X greater compared to patient samples that did respond (149). TWIST1 expression was also higher in an imatinib resistant CML cell line compared to a sensitive cell line (149). Furthermore, knockdown or overexpression of TWIST1 in CML cell lines led to enhanced sensitivity and increased resistance to TKI treatment respectively (146, 149). These data strongly implicate TWIST1 in driving TKI resistance in CML, however the mechanism/s involved remains to be determined.

**Figure 6:**
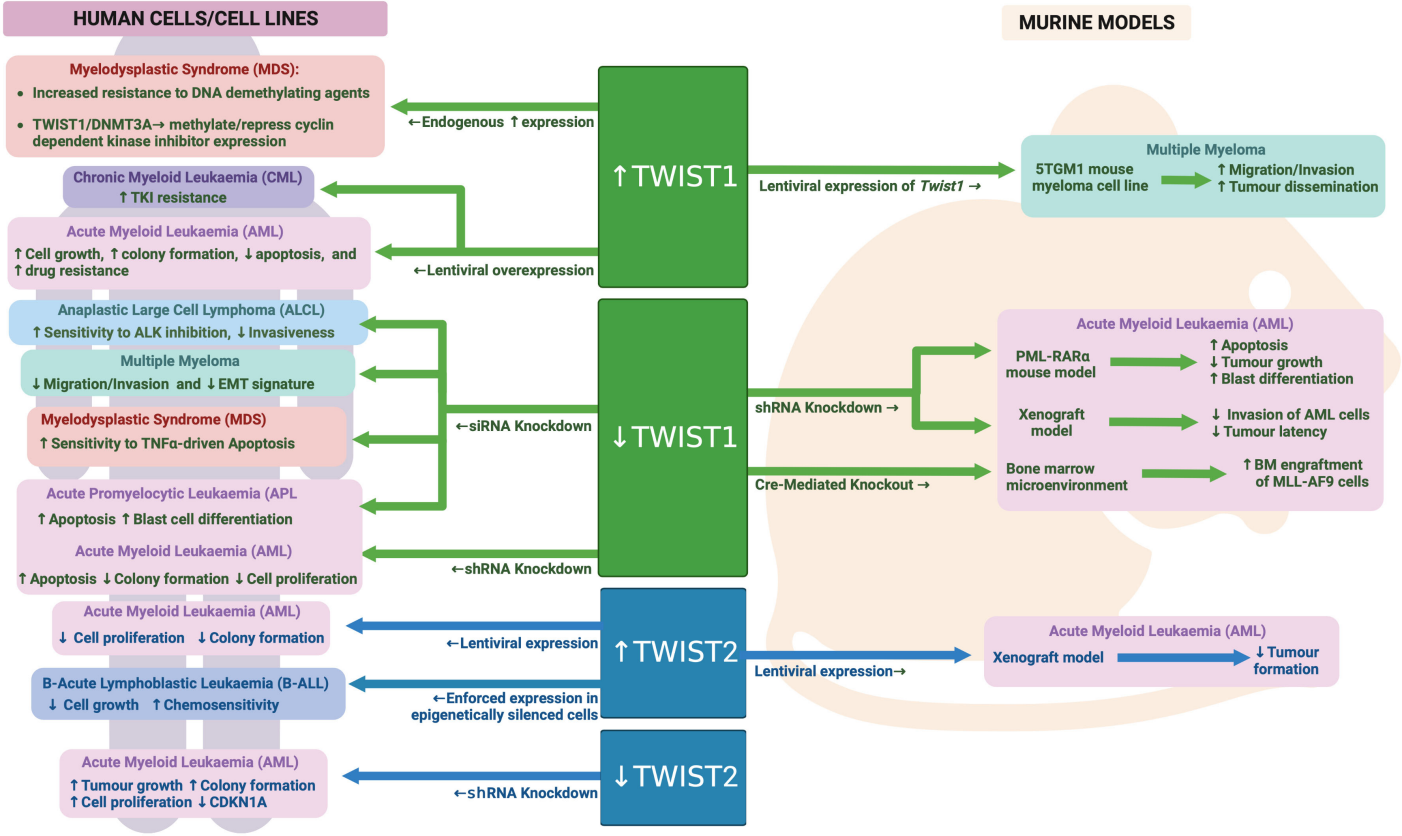
TWIST family during malignant hematopoiesis: Schematic outlining TWIST family roles in malignant hematopoiesis as determined through human and mouse model analyses. Created with BioRender.com.

While TWIST1 has been studied in much greater detail than its family member TWIST2 in the context of malignant hematopoiesis, the data currently available suggest opposing roles for these two proteins in AML. Whereas TWIST1 is upregulated in AML, TWIST2 is hypermethylated in ~30% of AML patients resulting in significantly reduced expression (150). Knockdown of TWIST2 in AML cells led to enhanced growth and colony forming capacity, while enforced TWIST2 expression in AML cells inhibited their growth and clonogenic capacity as well as protected mice from AML in a subcutaneous xenograft model. Mechanistically, TWIST2 expression was found to repress a number of known tumor suppressor genes as well as directly activate expression of the cell cycle regulator CDKN1A. Interestingly TWIST2 was not able to alter expression of known TWIST1 targets in AML, such as BMI1, suggesting different interacting partners and/or DNA binding sites for these two family members in AML cells (150). See **Figure 6** for an overview of TWIST family in malignant hematopoiesis.

### 5.3 Snail family

In keeping with the findings for ZEB proteins and TWIST1, SNAI1 is also highly expressed in AML cells compared to normal HSPCs (96, 139, 151), and is associated with worse overall survival (96) and chemotherapeutic resistance (151). To study the role of SNAI1 expression in AML, Carmichael et al. generated hematopoietic restricted SNAI1 transgenic mice. These mice all developed a myeloproliferative phenotype, which could transform into AML after a long latency of 12 months or greater (96). Analysis of *Snai1* transgenic mice at the pre-leukemic stage identified a significant skewing toward granulocyte/macrophage lineage development, with increased numbers of immature myeloid cells possessing increased self-renewal and mildly impaired differentiation capacity (96). Mechanistically, this SNAI1-driven hematopoietic phenotype was dependent on the histone lysine demethylase, LSD1, with physical interaction between the two proteins leading to impaired LSD1 function, altered DNA binding and aberrant target gene regulation. HSPCs ectopically expressing SNAI1 subsequently displayed altered gene expression programs related to normal myeloid differentiation, cytokine signaling, migration/invasion/adhesion and inflammatory pathways (96).

These findings suggest that hematopoietic restricted SNAI1 expression can predispose to malignant transformation of hematopoietic cells but does not directly drive it. Interestingly, Perez-Mancera et al. found that expression of a tetracycline regulatable *Combi-tTA*-*Snai1* transgene was able to induce tumor development in mice from 5 months onwards, with 40% of tumor being AML and 50% being lymphomas (152). The earlier onset and greater penetrance of AML development in the *Combi-tTA-Snai1* mice, as well as the lack of lymphoma formation in the hematopoietic-restricted model generated by Carmichael et al., suggest that either expression level differences between the two models (which is unknown at this time) or the non-hematopoietic expression of transgenic SNAI1 in the *Combi-tTA-Snai1* mice contributes to AML and/or lymphoma development.

SNAI2 expression is also significantly increased in human AML samples compared to normal bone marrow (153). This increased expression may be directly driven by AML oncogenes, as SNAI2 was found to be significantly upregulated in HSCs following viral transduction with *MLL-AF9*, *MEIS1* or *HOXA9* oncogenes. Furthermore, *Snai2* knock out was able to reduce the ability of *MLL-AF9* and *NUP98-HoxA9* oncogenes to transform mouse HSCs *in vivo*, while limiting dilution assays demonstrated reduced LSC/LIC frequencies in *Snai2* knockout MLL-AF9 leukemia. Homing of *Snai2* deficient MLL-AF9 AML cells was normal, however increased apoptosis and impaired cell cycle progression were apparent. These data suggest that upregulation of SNAI2 is important for the transforming ability of AML oncogenes (153).

Zhang et al. further confirmed these data in human AML, with *SNAI2* knockdown in AML cell lines resulting in reduced proliferative capacity and reduced LIC/LSC frequency. Use of a cell permeable peptide (TAT-SNAG), predicted to interfere with SNAI2 protein-protein interactions mediated by the SNAG domain, was also able to impair AML cell growth and colony formation as well as synergize with Cytarabine treatment *in vitro* to induce AML cell death. It is important to note, however, that the SNAG domain is highly conserved across SNAI family members as well as the GFI family of hematopoietic transcription factors (154). Therefore, this TAT-SNAG peptide may also inhibit the function of other SNAG-domain proteins and so these particular results cannot be conclusively linked to inhibition of SNAI2.

**Figure 7:**
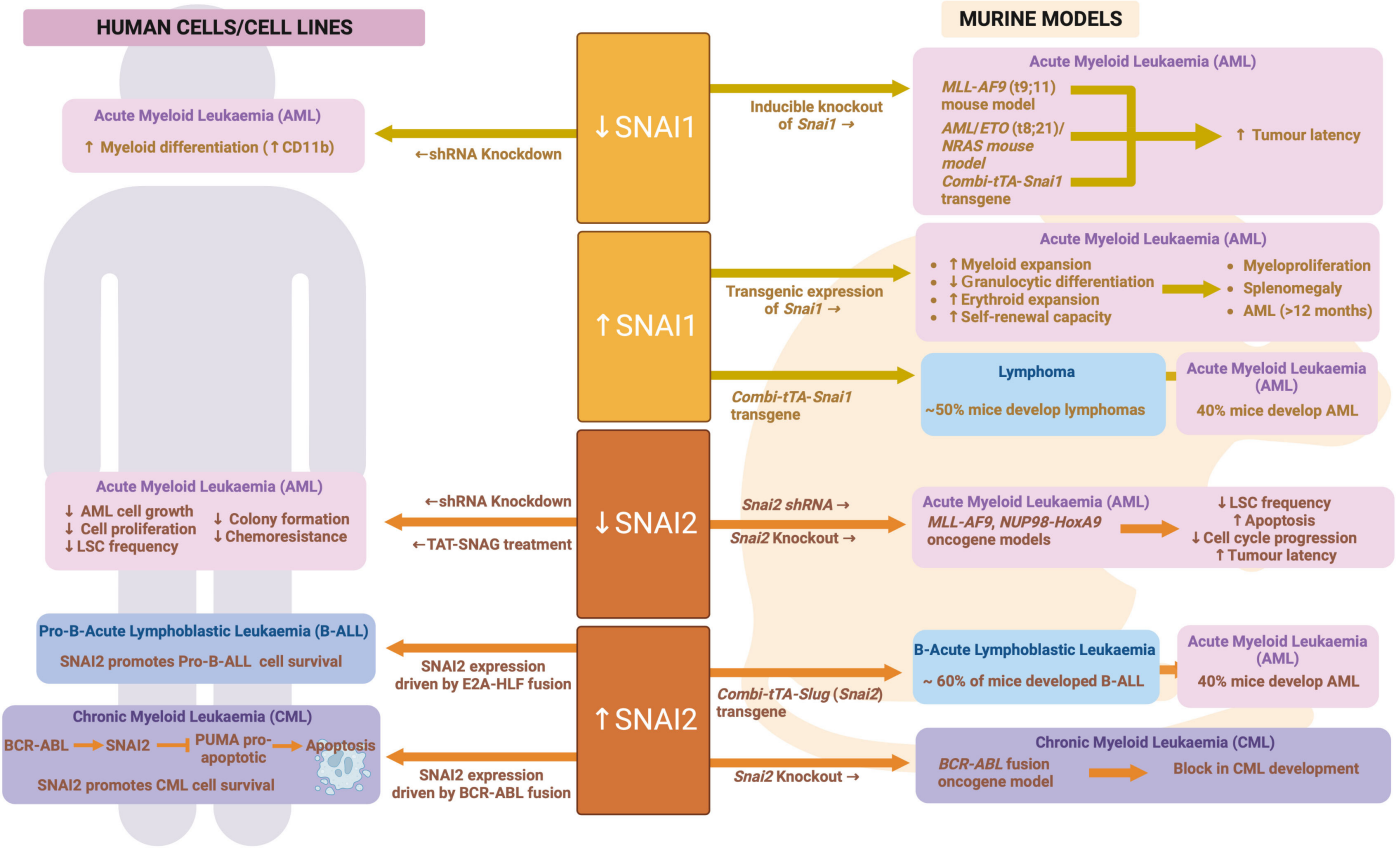
SNAIL family during malignant hematopoiesis: Schematic outlining SNAIL family roles in malignant hematopoiesis as determined through human and mouse model analyses. Created with BioRender.com.

As with SNAI1, a similar *Combi-tTA-Slug* (*Snai2*) transgene model was generated by Perez-Mancera et al. and also found to drive development of Acute Leukemias, of which 40% were AML (the other 60% being B-lymphoid) (97). Perez-Mancera et al. subsequently went on to show that expression of SNAI2 is upregulated in CML patient cells as compared to normal controls and is directly upregulated by the *BCR-ABL* fusion oncogene that drives the majority of CML cases. Strikingly, knockout of *SNAI2* was able to completely block CML development in a *BCR-ABL* transgenic mouse model, suggesting a key role for SNAI2 expression downstream of BCR-ABL (97). Furthermore, SNAI2 overexpression driven by BCR-ABL was shown by Mancini et al. to be reversed upon TKI treatment, leading to a release of SNAI2 driven repression of the pro-apoptotic protein PUMA and subsequent induction of apoptosis. In contrast, in CML samples carrying a TKI resistant BCR-ABL mutation, SNAI2 expression was even more enhanced than in TKI sensitive samples and helped to drive cell survival in response to TKI (155). These data demonstrate a clear role for oncogene driven SNAI2 expression in driving CML cell survival and therapy resistance. It is likely that similar mechanisms are at play in AML, where AML oncogenes can also upregulate SNAI2 expression. See **Figure 7** for an overview of SNAIL family in malignant hematopoiesis.

## 6 Lymphoid malignancies

### 6.1 Zeb family

Enhanced expression of ZEB2 has been identified in patients with early T-cell precursor Acute Lymphoblastic Leukemia (ETP-ALL), a particularly poor outcome subtype of T-ALL. Goossens et al. discovered a novel *BCL11B-ZEB2* fusion in rare cases of ETP-ALL, which is thought to drive increased ZEB2 expression through the 5’ BCL11B fusion partner (156). In other ETP-ALL cases, high expression of ZEB2 may result from downregulation of miR200c, a microRNA known to suppress ZEB2 protein expression (156). A direct functional link between ZEB2 expression and ETP-ALL was clearly demonstrated by the development of an ETP-ALL like disease in hematopoietic-restricted *Zeb2* transgenic mice from 5 months of age (156, 157). This same group also discovered that ZEB2 could physically interact with LSD1 in transgenic ETP-ALL cells, and intriguingly this interaction appeared to infer sensitivity to LSD1 inhibition (158). This sensitivity was further confirmed in human ETP-ALL cell lines with high ZEB2 levels, but not found in T-ALL lines without high ZEB2. A recent follow-up study by this group identified upregulation of the IL7R in *Zeb2* transgenic ETP-ALL cells as driving IL7-mediated activation of JAK/STAT signaling and upregulation of the pro-survival protein BCL2. This ZEB2/LSD1 interaction appears to repress pro-apoptotic genes such as BIM, making ETP-ALL cells susceptible to combined treatment with LSD1 inhibitor and the BCL2 inhibitor ABT-199 or the JAK/STAT inhibitor Ruxolitinib (159).

ZEB2 has also been implicated in B-cell Acute Lymphoblastic Leukemia (B-ALL) with likely pathogenic mutations identified in a small proportion of B-ALLs (160–162). Interestingly, in one study these *ZEB2* mutations were associated with a significantly worse overall survival and an increased likelihood of relapse, being found in nearly 30% of relapsed cases compared to only 2-3% of diagnosed cases (161). However, the significance of these results remains to be confirmed as the authors’ own subsequent work found this link to be less evident in a second cohort of patients. It is still unclear how the identified mutations affect ZEB2 function and how mutant ZEB2 contributes to B-All pathogenesis, however these mutations do appear to be associated exclusively with the “B-other” ALL subtype, which lacks common B-All associated fusion proteins such as BCR-ABL1, ETV-RUNX1 and MLL-fusions (163).

Interestingly, while ZEB2 expression is upregulated in ETP-ALL, ZEB1 appears to be reduced suggesting opposing roles for these two family members in this disease. The *LMO2* oncogene, which is specifically associated with the ETP-ALL phenotype, can directly repress *ZEB1* at the transcriptional level, and ZEB1 expression is negatively correlated with LMO2 expression in ETP-ALL cells. LMO2 can also physically interact with ZEB1 and block its DNA binding ability (164, 165). Wu et al. provided additional evidence to suggest that downregulation of ZEB1 is essential for the LMO2 driven stemness phenotype in T-ALL cells as well as resistance to methotrexate treatment, a chemotherapeutic drug used to treat T-ALL (165).

ZEB1 is also downregulated in other malignant T-cell diseases, specifically Adult T-cell Leukemia/Lymphoma (ATLL), driven by infection with HTLV-1, and cutaneous T cell lymphoma (CTCL). In ATLL, Hidaka et al. discovered that the *ZEB1* gene is frequently impacted by focal deletion of the 10p11 chromosomal region (~1/3 of cases) (84). However, other epigenetic mechanisms also likely lead to reduced ZEB1 expression in ATLL, as demonstrated by the ability of demethylating and deacetylating agents to restore ZEB1 expression in ATLL cell lines lacking a 10p11 deletion. A direct functional link between ZEB1 downregulation and ATLL development is evident from *ZEB1* mutant mice, which develop a CD4^+^ ATLL from as early as 3 months of age (84). In CTCLs, which consist of Mycosis Fungoides (early stage disease) and Sezary Syndrome (late stage disease), up to 65% of patients display focal deletion or somatic inactivating mutations in the *ZEB1* gene. A clear pathogenic role for these mutations in CTCL, however, has yet to be elucidated.

In contrast to T-cell malignancies, ZEB1 expression is increased in B-cell malignancies, specifically Mantle Cell Lymphoma (MCL) and Diffuse B Cell Lymphoma (DLBCL). Sanchez-Tillo et al. identified ZEB1 protein expression in 50% of MCL cases studied histologically, and found it to be directly correlated with b-catenin expression (166). ZEB1 expression was subsequently found to be driven by activated WNT-signaling in MCL cell lines, and was linked to enhanced proliferation, reduced apoptosis and resistance to chemotherapy (166). Expression of ZEB1 in MCL cells also enhanced their lymphoma spheroid growth potential and increased their resistance to Bortezomib – suggestive of a cancer stem cell promoting role for ZEB1 in MCL (167). High ZEB1 expression has also been observed in DLBCL patient samples, both through immunohistochemical staining (168) and qRT-PCR analysis (169). Lemma S et al. further determined that high nuclear ZEB1 expression is associated with adverse three year overall survival (168), while Zhao et al. linked ZEB1 expression with increased immune evasion of DLBCL cells *via* a feedback loop involving ZEB1/SNHG14/miR-5590-3p that ultimately drives upregulation of PD-L1 expression (169).

### 6.2 Twist family

Thus far, TWIST1 expression has not been investigated in the context of T- or B- ALL, however it is significantly expressed in CTCL (170–172). Increased TWIST1 expression appears to be due to either gain of the chromosomal region 7p21 (171) or promoter hypomethylation (172). Goswami et al. further determined that TWIST1 expression in CTCL increases with disease stage from the more indolent Mycosis Fungoides stage through to the advanced Sezary syndrome stage (173). TWIST1 is also upregulated in ALK+ Anaplastic Large Cell Lymphoma (ALCL), a common pediatric lymphoma driven by the t(2;5) NPM-ALK fusion. TWIST1 knockdown in ALK+ ALCL cell lines reduced their invasiveness and enhanced their sensitivity to an ALK inhibitor, suggesting TWIST1 may contribute to therapeutic resistance (174).

TWIST1 has also been implicated in Multiple Myeloma (MM). In ~15% of MM patients the t(4;14) translocation leads to enhanced expression of the NSD2 gene (175). Gene expression profiling by Cheong et al. identified EMT gene signatures correlated specifically with NSD2 high MM patient samples. They further demonstrated that TWIST1 expression is upregulated in t(4;14) MM cell lines but not in MM cell lines lacking this fusion. Knockdown of TWIST1 in NSD2+ MM cell lines led to downregulation of the EMT gene signature and reduced invasiveness *in vitro*. Conversely, enforced TWIST1 expression in a mouse MM cell line enhanced its migration *in vitro* and its dissemination/invasiveness *in vivo*, but did not impact on overall tumor growth and proliferation (176).

Promoter hypermethylation of the *TWIST2* gene is frequently observed in both childhood and adult ALLs (both B and T lineage) and is associated with loss of TWIST2 protein expression. Interestingly, while *TWIST2* hypermethylation was found in approximately half of diagnostic ALL cases, it was present in nearly all relapsed samples analyzed - suggesting a role for reduced TWIST2 expression in disease relapse and therapy resistance. Indeed, enforced expression of TWIST2 in B-ALL cell lines led to reduced cell growth and increased sensitivity to chemotherapy (177). *TWIST2* hypermethylation has also been observed in some chronic lymphocytic leukemia (CLL) patients, however no further investigation has been performed into possible associations or implications (177, 178). Similarly to TWIST1, TWIST2 was also found to be expressed highly in a Sezary syndrome (CTCL) cell line compared to a T-ALL cell line in one study, however no further evidence to support a role for TWIST2 in this disease has been published to date (129).

### 6.3 Snail family

To date, no studies have looked specifically at SNAI1 in lymphoid malignancies, however the *Combi-tTA-Snai1* transgenic mice do develop lymphomas in 50% of cases suggesting SNAI1 should be considered in the context of human lymphomas as well (152). SNAI2 was originally identified as a downstream target of the t(17; 19) E2A-HLF oncoprotein in human pro-B-ALL (179) implicating it in this disease. Concordantly, Inukai et al. found SNAI2 to be expressed in B-ALL cells expressing the E2A-HLF oncoprotein and their preliminary studies suggested SNAI2 plays an anti-apoptotic role downstream of this oncogene (179). Furthermore, in the *Combi-tTA-Slug* transgenic mice, 60% of the leukemias that developed were B-cell derived (97). These same authors further found SNAI2 to be highly expressed in cell lines and samples from B-ALL patients, however it remains unclear exactly how SNAI2 expression contributes to B lineage transformation.

## 7 Conclusion

The importance of EMT-TFs during hematopoietic development and their subsequent contribution to malignant hematological disease is an emerging area of research. The ZEB, TWIST and SNAIL families play distinct *and* overlapping roles throughout hematopoiesis, including regulating HSC self-renewal, quiescence and survival as well as differentiation along various myeloid and lymphoid lineages. The functions of EMT-TFs in hematopoiesis seem to be largely separate from the classical EMT processes they control during development. Instead, they regulate the expression and/or activity of key hematopoietic transcription factors, epigenetic modifiers, cytokine signaling pathways and regulators of cell survival and apoptosis.

In myeloid malignancies, increased expression of EMT-TFs has been identified and linked to worse overall survival and poor therapeutic response. In lymphoid malignancies, they have been implicated in disease development through either increased or decreased expression as well as mutations, deletions or fusions. Pathologically, in leukemia and lymphoma EMT-TFs contribute to enhanced LSC self-renewal and resistance to apoptosis, augmented tumor cell invasion and dissemination as well as the aberrant differentiation, cell growth and proliferation of tumor cells. It remains unclear, however, exactly how coordinated and discrete expression of these EMT-TFs is regulated during malignant transformation of hematopoietic cells, as well as what determines their oncogenic or tumor suppressive roles in different hematopoietic contexts.

It is intriguing to speculate about a potential role/s for EMT-TFs in regulating the immune response to cancer. While EMT-TFs have been implicated in controlling the cancer immune microenvironment from a cancer cell perspective, they may also play an intrinsic role in regulating the immune cells themselves. It is clear that EMT-TFs contribute to the normal differentiation, development and function of immune cells such as macrophages, DCs and T lymphocytes. They also regulate the expression of various inflammatory cytokines and chemokines, as well as genes involved in DC and T-cell activation. How immune cell intrinsic functions for EMT-TFs may contribute to cancer development, progression and outcome remains an important future question to address.

Despite significant progress in understanding the role of EMT-TFs in blood cell development and malignant transformation, there is still much to uncover about their complex mechanisms of action as well as their future promise as therapeutic targets. Further research in this area has the potential to reveal new insights into the underlying biology of leukemia and lymphoma and to identify novel approaches for the treatment of these aggressive hematological diseases.

## Author contributions

KM, LT and CC wrote the paper, reviewed and approved the final version.
